# Lung Dysfunction and Systemic Inflammation: A Role for HO-1 and NLRP3 in a COVID-19 Murine Model

**DOI:** 10.21203/rs.3.rs-8844647/v1

**Published:** 2026-03-17

**Authors:** Sophia Kwon, Joanna Zhou, Jamie Antelo Rivero, Hailey Bernier, Gabriele Grunig, George Crowley, Anna Nolan

**Affiliations:** New York University

**Keywords:** COVID-19, Airway hyperreactivity, lung injury, noninfectious translational model

## Abstract

**RATIONALE.:**

The COVID-19 (C19) pandemic caused significant mortality often due to lung injury and systemic inflammation, but there is significant heterogeneity in severity and the pathobiology is not well understood. We examined COVID-19-induced pulmonary and inflammatory sequelae using a murine noninfectious model to further define the models utility and to also understand the role of mediators such as heme oxgenase-1.

**METHODS.:**

k18-hACE2 male mice oropharyngeally aspirated C19-spike or equal volume control. After 72 hours, we collected: pulmonary mechanics, bronchoalveolar lavage(BAL) and plasma, snap-froze right lung, and fixed/stained left lung for histologic injury assessment(Qupath). Cytokine elaboration in BAL and plasma was quantified(Luminex), and lung homogenates were probed for HO-1 and NLRP3 (Western). Statistical (SPSS and R) and pathways comparisons(Ingenuity Pathway Analysis) were made between control and C19.

**RESULTS:**

**Lung Mechanics.** C19 exposure significantly reduced inspiratory capacity and static lung compliance;tissue elastance and airway hyperreactivity were increased. **Histology:** C19 exposure caused significant inflammation and thickened alveolar septae. **Cytokines:** C19 exposure led to inflammatory response in BAL and plasma with simultaneous activation of Type 1 and Type 2 pathways. **Pathways.** NLRP3 and HO-1 protein expression is significantly induced by C19. Regulator networks show involvement of multiple cell lines and lung damage.

**CONCLUSION:**

A noninfectious C19 murine model showed worsened lung parameters and increased inflammation. HO-1 and NLRP3 may be key mediators in the inflammatory process and induce both inflammatory and counter-regulatory effects. Further studies will focus on targeted therapeutic pathways that probe into the mechanistic relationship of HO-1 and NLRP3 in C19-related disease.

## BACKGROUND

At the end of the public health emergency of the COVID-19 (C19) pandemic in May 2023 by the World Health Organization), there were over 766 million cases worldwide, with 1.13 million deaths in the United States alone.^1^ C19 mortality is often attributed to inflammation, severe respiratory failure due to lung injury, catastrophic cascade of immune-mediated inflammation, and metabolic derangement.^2–11^ However, the associated morbidity and mortality of C19 is quite heterogeneous. Therefore, further phenotyping and characterizing murine models of C19 induced disease may foster the identification of mechanistic pathways and biologically plausible therapeutic targets that may prevent future pandemics.

Our laboratory has studied lung injury due to infectious and exposure-related causes such as particulate matter (PM).^12–18^ Murine models exposed to live virus have demonstrated several physiologic sequelae of C19 infection, including acute lung injury, asthma, and hyperinflammatory response.^19–24^ However, the high risks of infection and potential for mutation of a zoonotic disease requires rigorous, resource-intensive protocols and advanced BSL3 facilities to limit unintended infections. Moreover, the use of live virus models may restrict the ability to obtain more accurate lung function measurements, as invasive methods are often required instead of whole-body plethysmography.^19,20^ Among the emerging virus-free methods of exposure for pre-clinical studies, spike protein has emerged as a key method of inducing acute lung injury in a murine model.^25,26^ Spike protein offers the ability to easily modify experiments to account for different strains. Additionally, spike protein induces a multi-organ dysfunction in mouse models that include hyperinflammatory response, acute lung injury, ARDS, and long-term cognitive dysfunction.^25–27^ Therefore, this work intends to utilize a non-infectious model to further validate its utility, expand phenotyping, characterize associated pathways and biologically plausible targets.

One such target is Heme Oxygenase-1 (HO-1). HO-1 is the inducible isoform of HO and is vital in protecting the lung against oxidative damage.^28^ HO-1 expressed in the lung is upregulated upon oxidant-induced lung injury.^28,29^ It also has roles to downregulate the nucleotide-binding domain, leucine-rich-containing family, pyrin domain-containing-3 (NLRP3) inflammasome in different models of murine lung inflammation.^30^ In response to stress, HO-1 catalyzes the breakdown of heme into iron, carbon monoxide (CO), and biliverdin, which have potent antioxidant, anti-inflammatory, and anti-apoptotic functions.^31–33^ However, recent studies have suggested potential worsened outcomes with HO-1, including murine models of pulmonary fibrosis that inhibit HO-1 show attenuated collagen deposition.^34^

A pilot study of a PM-exposed cohort with global metabolomic serum profiling showed that HO-1-related metabolites including mesobilirubinogen and L-urobilinin were lower in those with airway hyperreactivity (AHR) and lung injury.^35^ We have also shown preliminary work that HO-1 is attenuated in a murine model of high fat diet, and lower levels were associated with worse pulmonary function after particulate matter exposure.^36^ How HO-1 and its by-products function in C19 is less clear. HO-1 was associated with poor COVID-19 progress and outcomes.^37,38^ C19 increases free heme, driving cytokine storm, and may inhibit HO-1. In contrast, HO-1 genetic polymorphisms are linked to low baseline HO-1 levels and subsequent increased inflammation.^39^ Amongst the by-products, free iron can be used for microbial replication processes, which may promote tissue injury and secondary infections.^40^ Low-level CO has been used for its vasodilatory and anti-inflammatory properties in the treatment of tissue injury, lung disorders, and ARDS, but may also be a marker of inflammation and cytokine storm.^41^ C19 has also been shown to bind to biliverdin and its by-product bilirubin, which helps it evade antibody immunity.^42,43^

**Figure 1.**
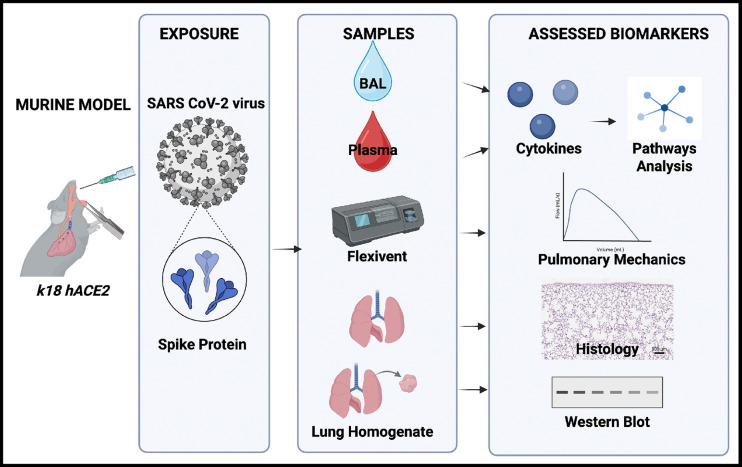
Overview of Murine C19 SP Non-Infectious Model Timeline and Analysis Pipeline

The mechanistic underpinnings of C19-associated disease and resultant mortality are not well understood, but furthering our understanding is key to developing targets for future interventions.^22,23,25^ Proposed mechanisms of triggered inflammatory state include NLRP3, caspase-1, and caspase-8.^44,45^ To address a critical gap in the current literature, we explore C19 spike protein exposure’s inflammatory effects on lung function, clinically relevant biomarkers, and perform functional pathway analysis, Fig. 1.

## METHODS

### Murine Model of C19 Spike Exposure.

Breeding pairs of heterozygous *K18-hACE2* mice (Strain B6.Cg-Tg(K18-ACE2)2Prlmn/J, Jackson Labs) had free access to standard chow/water and 12-hour light/dark cycles. All mice from each subsequent generation were genotyped by PCR as per vendor instructions and only males≥8 weeks old and ≥20g were used for subsequent experiments (Genotyping Core Laboratory, NYU). N = 30 male mice were utilized for pulmonary and biomarker assessment based on prior studies that showed pulmonary inflammation, and after preliminary studies using N = 5 female with C19 exposure had inconsistent results.^25^ Inclusion/exclusion criteria were applied and provided in **Supplemental Table 1**, and further explained in **Supplemental Methods.** All methods were performed in accordance with the relevant guidelines and regulations, including ARRIVE 2.0 guidelines (Animal Research: Reporting in Vivo Experiments; Essential 10) and the NYU IACUC (# 16–00447).^14,46–49^ In addition, all mice were euthanized at the end of the experiments by terminal dose of ketamine/xylazine, followed by confirmatory bilateral thoracotomy and exsanguination in accordance to the American Veterinary Medical Association Guidelines for the Euthanasia of Animals.^50^

SARS-CoV-2 spike protein (SP; RayBiotech), 400 μg/kg in 2 mL/kg body weight or control buffer in 2 mL/kg body weight total (RayBiotech) was administered via oropharyngeal aspiration as previously described, Fig. 1.^51–53^ This dose has been previously shown to induce acute lung injury similar in appearance to cases of acute C19 in humans 72 hours after exposure in a non-infectious model using male *K18-hACE2* mice.^25^ Littermates were co-housed and exposed to either control buffer or SP on the same day to avoid batch bias. 72 hours after SP or control exposure, mice received intraperitoneal (ip) anesthesia (0.12 ml/10g bodyweight of a mixture of ketamine (100 mg/ml, Covetrus) and xylazine (10 mg/ml, Troy Laboratories)), Fig. 1.

### Lung Mechanics.

N = 24 mice were tracheotomized with an 18g steel cannula for lung function and airway hyperreactivity measurements (methacholine challenge) (Flexivent; Scireq) as previously described.^14^ N = 12 mice were excluded for improper Flexivent measurements, but were potentially used for biomarker collection.

### Sample Collection.

Bronchoalveolar lavage (BAL) and plasma, via cardiac puncture, were collected immediately after lung function assessment. Lungs dedicated for histology were infused with 4% paraformaldehyde, fixed (at 25 cm of H_2_O pressure), mounted, electronically scanned, and assessed; (QuPath version 0.6.0).^54^ Lungs for protein assessment and/or Evans blue assessment were snap frozen in liquid nitrogen. BAL and plasma were thawed once and assayed (Cytokine/Chemokine Panel 1; Millipore Sigma) for 33 analytes.

### Immunoblots.

A portion of the right lung was lysed in NP-40 lysis buffer and probed for NLRP3 (Abcam AB 263899), MYD88 (Abcam AB2064), Caspase-1(Santa Cruz SC-392736), Caspase 8 (Santa Cuz SC-81656), RAGE (Santa Cruz SC-365154), and HO-1 (Abcam AB-189491) on a capillary based western blot (Wes, ProteinSimple). Each run was probed for beta-actin (Millipore Sigma, A2228) as a protein-loading control.

### Statistical Analysis.

SPSS 28 (IBM, USA), R Studio (Ver 2025.05.0 + 496), R (Ver. 4.5.1), and Graphpad Prism 10.4.1 (Boston, MA, USA) were used for database management and statistics. Continuous data was compared by Student’s t-test or Mann-Whitney U as appropriate. Data from multiplex biomarker assays were first analyzed in semi-supervised hierarchical clustering, and then PCA for data visualization. **Ingenuity Pathway Analysis (IPA) (Qiagen)** was used to assess upstream regulators, downstream effects, mechanistic and causal networks.^55^

Additional details may be found in the Supplemental Methods.

## RESULTS

### C19 SP Aspiration induces Acute lung injury and Airway Hyperreactivity.

**Figure 2.**
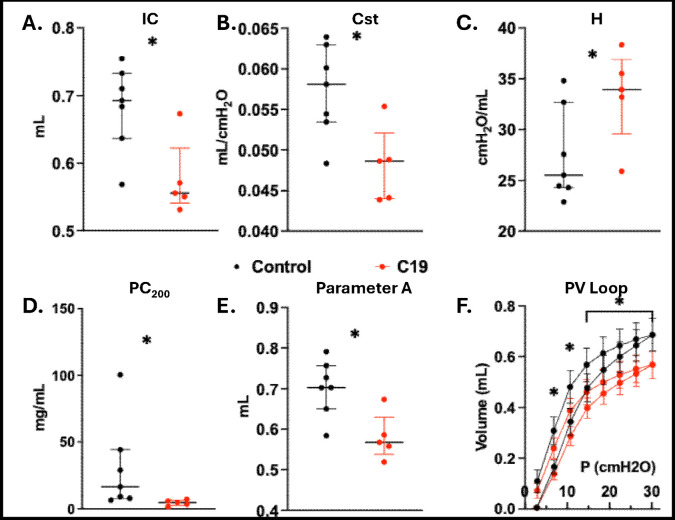
Lung Mechanics Assessment in C19 Spike Exposed Mice. **A.** Inspiratory capacity (IC) **B.** Static compliance (Cst) **C.** Tissue Elastance (H) **D.** PC_200_ showing more hyperreactivity after C19 exposure **E.** Parameter A relates to hysteresis of PV loop **D.** Pooled average Pressure Volume Curve of Control and C19, with significant differences in pressure at all points except baseline, N5 for each group.

We assessed the degree of C19-associated lung injury utilizing a comprehensive approach examining lung mechanics, proteinaceous leak, and histology. C19 mice (N = 5) had significantly lower inspiratory capacity (IC), static compliance (Cst), and increased tissue elastance (H) 72 hours after exposure compared to controls (N = 7), Fig. 2A–C. Moreover, C19 exposure induced airway hyperreactivity with lower PC_200_, Fig. 2D. Decreased Parameter A also affected hysteresis of the pressure-volume loop, compared to controls after 72 hours, Fig. 2E–F. There was no significant difference in other spirometry metrics including baseline resistance, **Supplemental Fig. 1**. C19 mice had an average of 25.50% (SD 14.91) macrophages on BAL cell count differential compared to controls 98.00% (1.79) macrophages, p = 0.01.

### C19 Induces multilobar inflammation.

**Figure 3.**
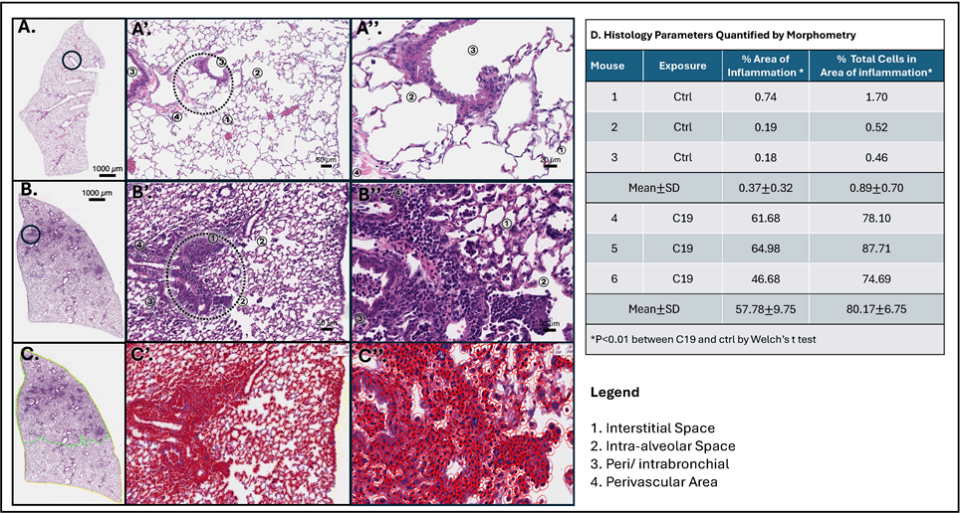
Histology and Measures of Lung Injury Post C19 Exposure. Representative histologic section of lungs of whole lung lobe with area of terminal airway at 1x further examined indicated by a circle for **A.** Control and **B.** C19 exposure. **A’.** Terminal airway of representative control at 10x and **A”.** 40x with clear interstitial space (1), intra-alveolar space (2), peri/intrabronchial space (3), and perivascular area (4), compared to **B’.** C19 exposed mice with evidence of focal inflammation visible at 10x and **B”.** 40x. **C.** Representative image of lung section analyzed on Qupath with entire lung highlighted in yellow and compared to areas of inflammation highlighted in green. **C’.** Terminal airway at 10x and **C”**. 40x with each identified cell and its nucleus outlined in red. Cells found in tissue and sequestered in the bronchoalveolar space are included in the final cell count. **D.** Table of histology parameters quantified by morphometry using Qupath of N=3 control vs N=3 C19 mice.

Mice with C19 exposure displayed more inflammatory changes on H&E stain compared to control mice. Representative images of pulmonary sections of buffer and C19-exposed mice are shown in Fig. 3A–B respectively. Compared to controls, C19 mice had patchy areas of inflammation, thickened alveolar septae, and inflammatory cell accumulation in the interstitial, intra-alveolar, intrabronchial, and perivascular areas, Fig. 3A’–3B’. There was also associated dense patches of inflammatory cells, collapse of the alveolar space, and focal atelectasis, Fig. 3A”–3B”.

A trained, blinded investigator (SK) annotated H&E stained sections of whole lung lobe (yellow) and areas of inflammation (green), Fig. 3C.^54^ Detected cells (red) were also identified using a threshold of 0.3 to detect alveolar and inflammatory cells, and compared in areas of inflammation to entire lung lobe, Fig. 3C’–C”. C19 (n = 3) had average of 57.78% of the lung lobe annotated as inflammation, compared to 0.37% in control mice (n = 3), p = 0.009, and 80.17% inflammatory cells compared to 0.89%, p = 0.002, Fig. 3D. Ratio of lung to plasma EB was not different in C19 exposure (n = 7, mean 88.46±SD 49.33) compared to control (n = 7, 63.69±SD 41.17), p = 0.32.

### C19 SP exposure yielded a differential BAL and Plasma cytokine biomarker signature and inflammatory profile.

**Figure 4.**
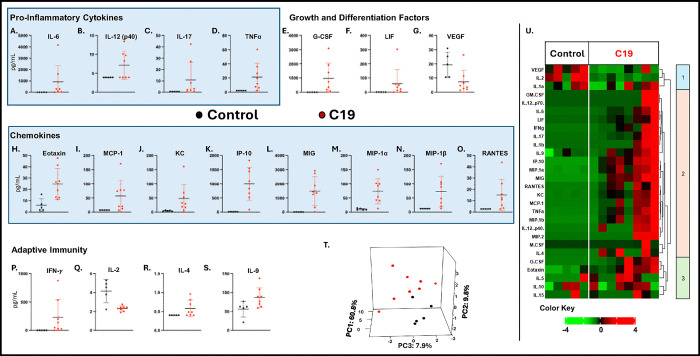
BAL Biomarker Assessment. C19 exposed mice had significantly higher expression of pro-inflammatory associated cytokines **A.** IL-6, **B. IL-12(p40) C. IL-17, and D. TNF-a.** Growth and Differentiation factors E. GCSF, and F. LIF, were increased after exposure, while G. VEGF decreased. Chemokines H. Eotaxin, I. MCP-1, J. KC, K. IP-10, L. MIG, M. MIP-1a, N. MIP-1b, O. RANTES were also increased. P. IFN-g was also increased, while Q. IL-2 was decreased. R. IL-4 and S. IL-9 increased after C19 exposure. **T**. **PCA** captured 78.5% of the total variance. **U. BAL Biomarker Heatmap** shows 3 main clusters showing different patterns of upregulation of many cytokines / chemokines in cluster 2 and 3, and downregulation of IL-2 and VEGF. IL-3, IL-7, IL-13, and LIX are excluded from the heatmap because all values were at minimum detectable concentration, N 5 for each group.

In **BAL**, C19 exposure significantly increased proinflammatory cytokines (Fig. 4A–D). Growth and differentiation associated factors G-CSF and LIF were increased after exposure to C19, while VEGF was decreased, Fig. 4E–G. Chemokines of multiple cell lines including eosinophils and neutrophils, such as Eotaxin and KC, were also elevated after C19 exposure, Fig. 4H–O. There was also activation of adaptive immune associated cytokines, with elevated IFN-γ, IL-4, and IL-9, but decreased IL-2, Fig. 4P–S. Other assayed analytes including pro-inflammatory cytokine MIP-2 and growth factor GM-CSF, were not significantly different between control and C19 in BAL, **Supplemental Fig. 2**. *PCA* using all measured analytes captured 78.5% of the total variance in 3 components, Fig. 4T. The heat map shows that VEGF and IL-2 segregated with lower expression in C19 in cluster 1, vs generally higher expression of the other inflammatory analytes in C19 exposure in cluster 2–3, Fig. 4U.

**Figure 5.**
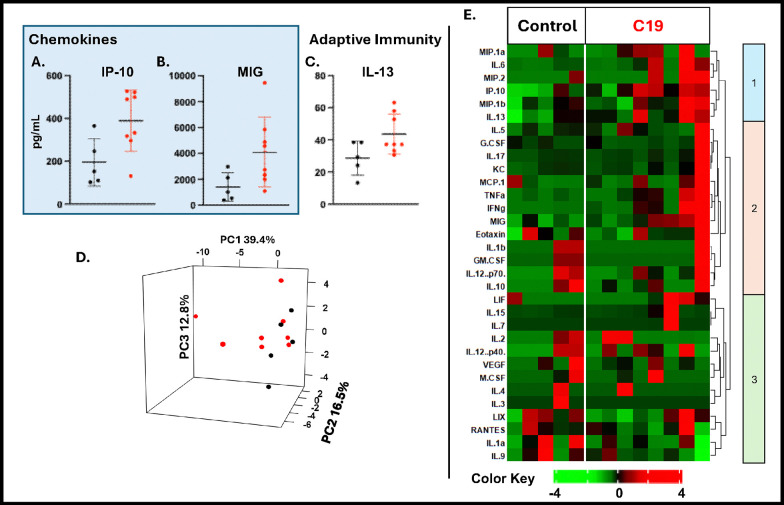
Plasma Biomarker Assessment. C19 exposed mice had significantly higher expression of **A.** IP-10 **B.** MIG and **C.** IL-13 **D. PCA** 68.7% of the variance was captured in 3 components. **E. Heatmap and Clustering** shows 3 clusters, with upregulation in C19 in cluster 1, equivalence in cluster 2, and downregulation in cluster 3.

In **plasma**, there was activation of both chemokines and the adaptive immune response-associated cytokines with IP-10, MIG and IL-13 significantly elevated in C19-exposed mice, Fig. 5A–C. All other assayed analytes can be seen in **Supplemental Fig. 3.** PCA captured 68.7% of the variance in 3 components, Fig. 5D. IL-13 and IP-10 segregated within Cluster 1, while MIG segregated to Cluster 2 in the heatmap, with both clusters associated with upregulation in C19-exposed mice, Fig. 5E. BAL and plasma analytes were also plotted by fold-change and p-value in volcano plots, **Supplemental Fig. 4A and B respectively**. In BAL, 17/32 analytes significantly increased in fold-change(Eotaxin, MIP-1α, MIP-1β, RANTES, G-CSF, KC, IL-10, MIG, IFN-g, IL-12(p40), IL-4, IL-6, LIF, MCP-1, and TNF-α) and decreased in IL-2 and VEGF, **Supplemental Fig. 4A**. In plasma, MIG was identified as significantly upregulated (p < 0.05) **Supplemental Fig. 4B**.

### Multiple Inflammatory Pathways are Activated in C19 Exposure

**Figure 6.**
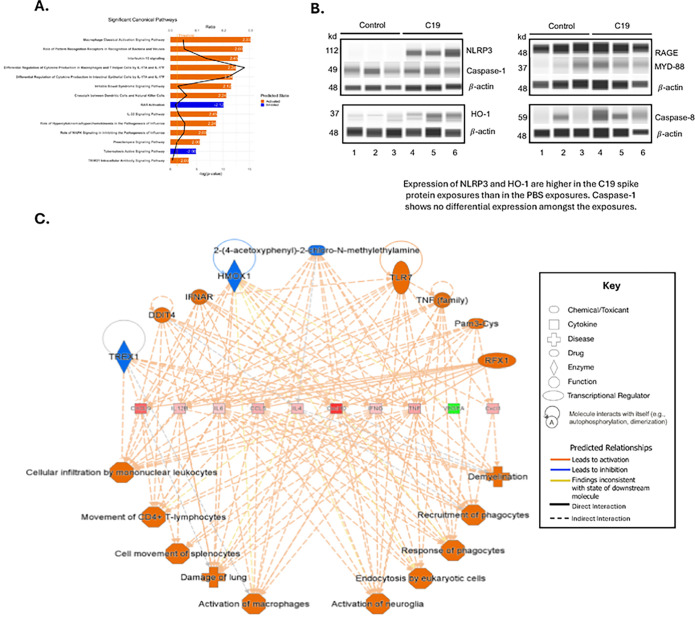
Pathways Assessment of the C19 affected Lung. **A. Significant BAL Canonical Pathways** (p-value threshold 0.05). Bars show top 14 activated (orange) and inhibited (blue) significant canonical pathways ranked by −log(p-value), with z-score >2 in white. Line (black) shows ratio of analytes in pathway **B. Immunoblots.** Control (Lanes 1–3) compared to C19 exposed (Lanes 4–6), probed for NLRP3, Caspase-1, HO-1, RAGE, MYD-88, Caspase-8, and b-actin housekeeping gene. Blots have been cropped for clarity and conciseness. Contrast has been optimized for visibility and has been equally applied for controls and C19 exposure for each target protein. **C. Regulator Effects Analysis** with Highest Consistency Score

Transformed BAL and p-value data was input into IPA, and n = 14/192 significant canonical pathways that met threshold with p-value < 0.05 also had |z-score|>2, Fig. 6A. Multiple inflammatory pathways were activated including macrophage, T-cell response (pattern recognition receptors), and natural killer cells. The top significant identified canonical pathway, Macrophage Classical Activation Signaling Pathway is shown, **Supplemental Fig. 5**.

NRLP3 and HO-1 were significantly induced in the C19 spike protein exposures compared to control, Fig. 6B. Caspase-1, Caspase-8, RAGE, and MYD-88 were not significantly different. Full Western images are provided in **Supplemental Fig. 6–9**. Additionally, a regulators effects analysis was performed in IPA, and the analysis that involved HMOX1, the gene that encodes HO-1 with the highest consistency score, a measurement used to help rank the most highly connected and consistent networks from regulator to function, is presented, Fig. 6C. Regulator networks showed recruitment of multiple cell lines including leukocytes, CD4 + T-lymphocytes, phagocytes, and macrophages, as well as damage of lung.

Upstream regulators that were significantly activated or inhibited (|z-score|>2) are also displayed for BAL and plasma, **Supplemental Fig. 10**. TreeMaps of disease and functions in BAL showed marked increased activity in multiple inflammatory pathway activation and cell recruitment pathways, **Supplemental Fig. 11**. In plasma, the significance threshold was not met to fully develop a TreeMap.

Full pathways analysis are available at: IPA_BAL and IPA Plasma.

## DISCUSSION

C19 overwhelmed hospitals globally with high numbers of patients with multiorgan failure, often with cytokine release syndrome, and dysregulation of the immune system.^56^ Future pandemics remain an ongoing threat, and it is critical to understand the mechanistic underpinnings of C19 to identify potential targets of future therapy. Our work contributes to the C19 literature by clarifying knowledge gaps validating a non-infectious murine body, quantifying lung function changes and end-organ dysfunction. Moreover, we have identified potential therapeutic targets by showing upregulation of both HO-1 and NRLP3. These targets are supported by our findings in IPA, showing the involvement of mediators central to HO-1 and NLRP3 activation.

This noninfectious transitional model recapitulated many of the findings found using noninfectious models of COVID, and significantly overlaps many of the phenotypes seen in live-virus models.^25,57^ Lung function decline after COVID infection was characterized by reduction in IC, compliance, volume, and hysteresis.^19^ Our model differed by additionally showing airway hyperreactivity. Further, despite significant involvement of lungs after C19 exposure, there was no alveolar leak or an ARDS phenotype.

Exposure to C19 induced a simultaneous elevation of several inflammatory and counterregulatory pathways in BAL, affecting Th1 (TNF-*α*, IL-6, IFN-*γ*, IL-12(p40), IL-2, IP-10, MIG), Th2-associated response (IL-4, IL-9, Eotaxin), and Th17 associated mucosal response (IL-17, KC, G-CSF). This could indicate a severe, overactive inflammation. The suppression of IL-2 and VEGF could indicate T-cell exhaustion and failed tissue repair.^58,59^ In plasma, although there were less significantly different inflammatory markers, there were similar patterns of mixed and often opposing immune signal from Th2 (IL-13), and Th1/interferon driven signaling (IP-10 and MIG) elevation after C19 exposure. There is similarity to cytokine release syndrome or cytokine storm seen in humans with C19, specifically in the elevation of IL-6, TNF-α, and IFN-γ.^60^ IL-1, which was also seen as critical to the cytokine release in humans, was measured as different isoforms in the murine model. Of those, IL-1β trended higher in C19-exposure. The multiple and sometimes counter-regulatory pathway involvement may also be contributory to the pathogen-induced cytokine storm signaling pathway initially being identified as the top related canonical pathway in IPA, but it was not significant by z-score.

We demonstrated that exposure to C19 spike protein can induce NRLP3 and HO-1, which may trigger downstream activation of inflammatory complexes. HO-1 is considered cytoprotective with antioxidant, anti-inflammatory, and antiviral properties reflective of activation of M2 macrophages.^37,61–63^ However, its upregulation has been associated with poor outcomes in C19-infected patients, specifically worse hypoxia and mortality.^37,61,64^ NLRP3 inflammasome activation in severe viral disease, such as in C19 and influenza, has also been thought to be at the center of immune-mediated dysregulation.^65–67^ It plays both pro-inflammatory activation roles and anti-viral response, but excessive stimulation has been linked to cytokine storm and multi-organ failure.^67^ In the lung, HO-1 activation is often seen to protect against the NLRP3 inflammasome via counterregulatory action in disease states such as cigarette-smoking induced COPD or LPS/sepsis-induced lung injury.^68–71^

Simultaneous activation of HO-1 and NLRP3 has been seen in chronic inflammatory disease such as osteoarthritis.^72^ The simultaneous activation of NLRP3 and HO-1 may indicate that there is activation of both inflammatory and counter-regulatory pathways, and point to possible chronicity with ongoing inflammation. This also concurs with the quantified cytokine profile of the murine model that showed upregulation of multiple immunoregulatory pathways.

The involvement of HO-1 and NLRP3 is also evident from the pathway and regulator analysis. Macrophage classical activation signaling pathway was the most significant canonical pathway. Heme was also the most significant inhibited upstream regulator in BAL. This could be related to macrophage activation by heme breakdown in the HO-1 pathway. Heme is also an important NLRP3 activator, and its breakdown may be early counterregulatory mechanism action against NLRP3.^73^ Further studies are needed to identify the complex mechanisms between HO-1 and NLRP3 activation in viral disease.

This study has several limitations. Male mice were used exclusively because preliminary studies using female mice had inconsistent data. One possible reason for this may be because male mice weighed approximately 30% more than the female mice, and received a higher overall dose of SP. Another reason may be because female mice exhibit hormones that influence the pulmonary inflammation profile differently from male mice. Future studies could investigate the use of a fixed, higher dose of SP to observe if there are any sex-related effects.

Our model differed in some aspects of lung dysfunction compared to both live and noninfectious models. There could be differences in live virus used compared to the noninfectious S1 spike protein.^19^ Our model may also need a longer time interval to show increased resistance as the pulmonary disease progresses, or a higher dose to induce more profound pulmonary changes. We also could not recapitulate vascular permeability measures of alveolar leak through either Evans Blue or electric cell-substrate impedance measurements.^25,26^ This could be due to small differences in technique of aspiration or injection of EB.

It is also possible that the inflammatory profile would change over time, and that 72 hours may not represent the optimal time for peak inflammation.^57^ Different doses and time intervals may have also shown different inflammatory profiles in not only the BAL, but also more peripherally in the plasma to represent more systemic inflammation. Moreover, it is possible that since this study did not use live virus, the inflammatory effects seen may not be generalizable to other strains of COVID or live virus studies. Nevertheless, this study represents an important step in studying C19-related inflammation using the isolated spike protein. The non-infectious model allowed us to measure the pulmonary changes that occur after exposure within a BSL-2 facility. There are also several aspects of the study that show moderate translatability of using a non-infectious model.

### Future Plans

This work adds to the growing body of literature aimed at assessing reproducibility and translatability of noninfectious models of C19. Further, it guides future studies using noninfectious models of C19 to investigate inflammatory response and lung injury. Future plans include loss and gain of function studies to identify NLRP3 and HO-1 as potential targets to mitigate inflammatory response in C19. Additional time-course and dose studies may also be studied for future studies to help profile the inflammatory progression. Noninfectious aspiration models of additional environmentally relevant irritants also may lay the foundation for future experiments investigating possible synergistic inflammation from other concurrent exposures.

## Supplementary Material

Supplementary Files

This is a list of supplementary files associated with this preprint. Click to download.

• 03Bsupplementalfig012725.pdf

• 03ASupplementalMethods.docx

• SupplementalTable1.docx

• SupplementalFiguresLegends.docx

## Data Availability

The datasets used and/or analyzed during the current study are available from the corresponding author on reasonable request.

## Abbreviations

AHR: Airway Hyperreactivity
ALI: Acute Lung Injury
ARDS: Acute Respiratory Distress Syndrome
C19: COVID-19
IACUC: Institutional Animal Care and Use Committee
IL: Interleukin
IP: Interferon gamma inducible protein
IRB: Internal Review Board
N: Number
NIH: National Institutes of Health
NYU: New York University
OAD: Obstructive Airways Disease
SP: Spike protein
TNF: Tumor Necrosis Factor

## Abbreviations related to Flexivent Parameters

Parameter A: relates to hysteresis of PV loop
Parameter K: relates to hysteresis of PV loop
Crs: Compliance of the Respiratory System
Cst: Static compliance
Ers: Elastance
FEV_0.1_: Forced expiratory volume in 0.1 second
G: Tissue Damping
H: Tissue Elastance
IC: Inspiratory Capacity
NPFE: negative pressure-driven forced expiratory maneuver
PC_200_: Dose of methacholine that doubles baseline resistance
Rrs: Resistance of the Respiratory System
Rn: Newtonian Resistance
